# Care need and dry mouth as risk indicators for impaired taste and smell

**DOI:** 10.1038/s41598-021-99978-3

**Published:** 2021-10-14

**Authors:** Mara-Zoe Hummelsheim, Stefanie Hamacher, Anna Hagemeier, Michael Johannes Noack, Anna Greta Barbe

**Affiliations:** 1 Department of Operative Dentistry and Periodontology, Centre of Dental Medicine, University of Cologne, Kerpener Str. 32, 50931 Köln, Germany; 2 Institute of Medical Statistics and Computational Biology, Medical Faculty, University of Cologne, University Hospital Cologne, Köln, Germany

**Keywords:** Medical research, Risk factors, Signs and symptoms

## Abstract

To identify whether reduced saliva secretion or xerostomia symptoms are risk indicators for impaired taste and smell, depending on age and care needs. This cross-sectional study evaluated taste and smell in patients categorized into different age groups (<65> years) and different care need, with and without dry mouth. Of the 185 patients included, 119 were classified as “dry mouth” and 66 as “without dry mouth”. Overall, 103 (55.7%) were female and 37 (20%) needed care. There was no difference between “dry mouth” and “without dry mouth” regarding identification of odors or tastes, but a difference in the number of correctly identified odors and tastes in favor of “without care need” patients (*p* < 0.05). The ability to identify smells and tastes was negatively influenced by age, number of medications, and number of comorbidities, but subjective dry mouth had no impact. According to our results, subjective dry mouth is not a risk factor for an impaired ability to recognize smells and tastes. However, care need representing age, the number of medications taken, and the number of chronic comorbidities is a risk indicator.

## Introduction

The older patient population, especially those in need of care, is the fastest growing patient group worldwide (United Nations, 2020). Older people are well known to be affected by subjective (xerostomia) and objective (hyposalivation) dry mouth problems^1^. These changes in salivary secretion are one of the most commonly-reported oral symptoms, with increasing frequency from middle to very old age, and negatively affect quality of life^2^. Salivary dysfunction can be divided into: (1) xerostomia (the subjective feeling of a dry mouth); (2) hyposalivation (an objective reduction in saliva secretion), and (3) changes in saliva composition^3^. Although xerostomia is frequently a manifestation of reduced salivary flow, it can also be a symptom on its own. Previous research has shown that xerostomia may also be related to changes in the biochemical composition of saliva or altered protein structure^4,5^. Dry mouth complaints can be described as a combination of subjective and objective complaints, possibly supplemented with functional and/or dental complaints resulting from the dry mouth. Hyposalivation also has multiple negative effects on oral health, including increased risk of caries, halitosis, prosthetic problems, an increased for candidiasis^6^ or burning mouth^7^. Dry mouth problems increase the risk of malnutrition, for example by exacerbating dysphagia or preventing optimal bolus formation when eating, with far-reaching consequences for general geriatric health^8–10^. The main cause of dry mouth seems to be the frequent use of medications and over-the counter-drugs, which increases with age^11,12^. Many of these drugs alone induce hyposalivation, but the ability to taste and smell may be affected when a number of these drugs are used concomitantly^13,14^; in turn, this can lead to changes in appetite and food intake. Impaired taste has been associated with increasing age^15,16^, diseases and drugs^17^, and dry mouth^18^.

Due to a large number of heterogeneous studies in different age groups, as well as differences in research methods regarding the diagnosis of dry mouth, little is known about the risk indicators for impaired smell and taste in different age groups, or whether subjective or objective dry mouth contribute, particularly in combination with other risk indicators such as the need for care.

Regarding the mechanisms behind functioning and impaired taste and smell abilities, the perception of aromas is seen in the literature as an integration of different sensory perceptions into a functional sensory system (“cross-modal perception”). Accordingly, taste stimuli and somatosensory impressions from the oral cavity and olfactory perceptions ensure the perception of an aroma, with the olfactory component most frequently defining the aroma identity. At the same time, retro-nasal smells seem to be more relevant than ortho-nasal ones. The most important brain areas for the integration of the various sensory impressions into an aroma impression are in the insula, the operculum, the orbito-frontal cortex, the anterior cingulate cortex, and the amygdala^19,20^.

Therefore, the correct functioning of many organ systems within the whole body system is involved in correct taste and smell. The number of diseases that may lead to taste and smell impairments are diverse and it is not easy to name a single cause. Different organ systems can be involved, which is why both the diagnosis and therapeutic options vary widely and can often only function in a multidisciplinary approach^21^. As described above, dry mouth and care need may represent these multifactorial limitations in many older people. We hypothesize that subjective or objective dry mouth problems and care need representing a high number of comorbidities and medications serve as risk indicators for the reduced abilities to taste and smell.

Our retrospective cross-sectional study evaluated differentiated taste and smell diagnostic data in patients categorized into different age groups and different care need categories, with and without subjective and objective reduced salivary output. Our objective was therefore to identify whether objective reduced salivary output or subjective dry mouth symptoms might serve as risk indicators for impaired taste and smelling, depending on age and care needs.

## Materials and methods

### Ethics and guidelines

The University of Cologne local ethics review board (19-1289) granted approval for the study. The study was registered under DRKS00024130 (date of registration: 25/01/2021) at the German Clinical Trials register (https://www.drks.de/drks_web/navigate.do?navigationId=resultsExt, lastly assessed 05/04/2021). All research was performed in accordance with relevant guidelines/regulations. This research has been performed in accordance with the Declaration of Helsinki and is reported according to the the STROBE guidelines^22^.

### Subjects

In this cross-sectional study, patient routine data were retrospectively evaluated. Data were taken from records of patients who had presented during 02/2018–08/2020 at the Department for Operative Dentistry and Periodontology, Cologne University Hospital, Germany, either for consultation regarding dry mouth problems or for participation in dry mouth intervention studies at the center. None of the patients who were included took part in therapy or intervention studies for saliva deficiency or symptom relief of dry mouth at least 12 months prior to the run-up to the study. The files from the named groups of people were scanned by two data extractors (HM and one person not otherwise involved in this study) regarding the inclusion criteria. All files that met these criteria were included. The records on the included patient cases were transferred from the electronic documentation software to case report forms (CRFs) and then transferred from the CRFs to the SPSS database. In this center, all patients gave their written consent that their data may be used for later retrospective pseudonymized data analysis.

### Clinical parameters

#### Oral examination

Decayed, missing, filled teeth (DMFT) index and periodontal situation documented by the community periodontal index of treatment needs (CPITN) were documented^23^. The root caries index (RCI) was graded on a scale from RC1 (hard surface) to RC5 (soft surface)^24^.

#### Xerostomic visual analogue scale (xVAS)

Participants were asked “How dry is your mouth?”, and answers were recorded as continuous variables from 0 = “not dry at all” to 10 = “no saliva at all”^25^.

#### Unstimulated salivation rates

All saliva collections took place between 9 and 11 a.m. in a quiet room in the Department of Operative Dentistry and Periodontology, Cologne, Germany. Due to the standard operating procedures of the department, participants did not consume any food or drinks 2 h prior to the examination and did not brush their teeth during this time. Participants were asked to relax for a couple of minutes before starting saliva collection. They were sat in an ordinary chair bent forward and asked to hold their mouth open and remain still, letting the saliva drip into a disposable cup held to the lower lips for 5 min. Volumes (ml) were determined using luer slip syringes (BD Discardit II, Becton, Dickinson and Company, Europe).

#### Stimulated salivation rate

The clinical collection of chewing-stimulated whole saliva samples has been outlined in detail^26,27^. In brief, stimulated saliva sampling was started by flushing in tap water followed by chewing on paraffin wax (Ivodent Vivodent AG, Liechtenstein) (1 g) for 30 s as a prestimulating procedure. Subsequently, while further chewing at a fixed frequency of 50–60 chewing cycles per minute, participants were instructed to spit every 60 s for 5 min in a sterile plastic cup to obtain the rate (ml/min); during the last few seconds of the 5 min, the resting amount of saliva also was collected. Volumes collected were determined using luer slip syringes (BD Discardit II, Becton, Dickinson and Company, Europe).

Although other methods to determine the volume of saliva might be more accurate (i.e., gravimetrically^28^), in this retrospective data setting we could not choose the diagnostic method. The described method is the method routinely used in the department of operative dentistry and periodontology.

### Sniffin’ sticks

Testing of smell was performed using the validated Sniffin’ Sticks test (Burghart GmbH, Wedel, Germany) with commercially available felt-tip pens^29,30^. According to the manufacturer's information for smell presentation, the cap of the pen was removed by the experimenter for approximately 5–20 s, and the tip of the pen was placed approximately 1–2 cm in front of the patient’s nose. Smell definition was evaluated using 12 common smells. The participants were asked to choose from a list of four possible definitive terms for each smell. Each smell compound was presented by the examiner and a pause of at least 30 s was provided to prevent desensitization to smell. No time limit was set on the patients. The test result was recorded per smell and as the total score of accurately identified smells.

### Taste strips

For objective determination of taste impairments, Taste Strips (Burghart GmbH, Wedel, Germany) were used. This validated test contains individual spoon-shaped strips impregnated with the tastes sweet, sour, salty, and bitter. Each strip was placed in the middle of the tongue and the mouth was closed. Subjects had to decide between the answers “no taste,” “sweet,” “sour,” “salty”, and “bitter.” After each taste strip, the mouth was thoroughly rinsed with water^31^.

### Data analysis and sample size calculation

In this retrospective study on the generation of hypotheses, all possible patients were included based on a previously-defined period according to the inclusion and exclusion criteria. As the aim was not to randomize according to a sample size estimate, the *p* values provided should be regarded as exploratory. Data were analyzed descriptively: absolute and relative frequencies are given for qualitative variables and mean ± standard deviation for quantitative variables for reasons of comparability with other works in the literature. Medians and interquartile ranges were also reported. The patient data sets were divided into the groups “dry mouth” (with subjective or objective (or both) dry mouth symptoms) and “without dry mouth” (absence of both subjective and objective dry mouth problems). Patients were also grouped according to “with care need” and “without care need”, based on the nursing grade. Group differences were tested using unpaired t-test or Fisher’s exact test, respectively. Univariate linear regression models represented as regression coefficient with an associated 95% confidence interval and *p* value were performed for single odors and tastes. All reported *p* values are two-sided and considered statistically significant if lower than 5%. All calculations were done with SPSS Statistics 26.0.0.1 64-Bit (IBM Corp., Armonk, NY, USA). Data were entered twice and reconciled in case of inconsistencies.

### Informed consent

Informed consent was obtained from all subjects in this study.

## Results

### Clinical characteristics

We included 185 datasets: 119 (64%) with “dry mouth” and 66 (36%) “without dry mouth”. Overall, 103 (55.7%) were female and 37 (20%) needed care (Table 1). The oral examination showed a mean DMFT of 18.5 ± 6.4, 107 (58%) suffered from periodontitis (CPITN), and the mean RCI was 0.3 ± 0.7. The mean age was 66.2 ± 15.1 years. Periodontitis (*p* = 0.034), RCI (*p* = 0.016), and the total number of chronic diseases (*p* = 0.013) were significantly higher in “dry mouth” patients than those “without dry mouth”. “Dry mouth” patients reported a mean VAS of 5.6 ± 2.7, mean whole unstimulated salivary flow rates of 0.3 ± 0.3 ml/min, and mean whole stimulated flow rates of 0.8 ± 0.8 ml/min. Chronic conditions are shown in Table 2.

**Table 1:** Clinical characteristics.

	Total no. participants (N = 185)	“Without dry mouth” (n = 66)	“With dry mouth” (n = 119)	*p* value*
**Number of patients, n (%)**				
Gender, female	103 (55.7)	36 (54.5)	67 (56.3)	0.878
Care need	37 (20)	15 (22.7)	22 (18.5)	0.566
Periodontitis (CPITN)	42 (57.5)	11 (84.6)	31 (51.7)	**0.034**
Hyposalivation				
Unstimulated	56 (31.6)	0 (0)	56 (49.5)	**< 0.001**
Stimulated	63 (36.6)	0 (0)	63 (55.8)	**< 0.001**
**Mean (SD) or median (IQR)**				
Age, years	66.2 (15.1)	65.7 (13.8)	66.5 (15.8)	0.578
	67 (57–78)	65.5 (57–77)	68 (57–78)	
Time in nursing home, months	19.8 (14.7)	23.9 (17)	17.2 (12.8)	0.264
	16.5 (7–31)	23 (15–33)	14 (5–29)	
Total API, n	3.4 (3.5)	3.1 (3.8)	3.6 (3.3)	0.095
	2 (1–5)	2 (0–5)	2 (1–5)	
Chronic diseases, n	2.5 (2.5)	2.1 (2.5)	2.7 (2.6)	**0.013**
	2 (1–3)	1 (0–3)	2 (1–3)	
BMI	25.5 (4.5)	25 (3.6)	25.9 (5.2)	0.366
	25 (22–28)	24 (22–27)	25 (23–28)	
DMFT	18.5 (6.4)	17.8 (6.4)	19 (6.3)	0.227
	20 (15–23)	19 (14–22)	20 (15–23)	
XVAS	3.8 (3.3)	0.5 (0.9)	5.6 (2.7)	**< 0.001**
	4 (0–7)	0 (0–0.5)	6 (4–8)	
Salivary flow rate, ml/min				
Unstimulated	0.4 (0.4)	0.6 (0.4)	0.3 (0.3)	**< 0.001**
	0.3 (0.2–0.5)	0.5 (0.4–0.7)	0.2 (0.1–0.4)	
Stimulated	1.2 (0.9)	1.8 (0.8)	0.8 (0.8)	**< 0.001**
	1 (0.4–1.6)	1.6 (1.1–2.4)	0.6 (0.3–1.2)	
Root caries index	0.3 (0.7)	0.2 (0.6)	0.3 (0.7)	**0.016**
	0 (0–0.2)	0 (0–0)	0 (0–0.2)	

*API* active pharmaceutical ingredients, *BMI* body mass index, *CPITN* community periodontal index of treatment needs, *DMFT* decayed missed filled teeth, *IQR* interquartile range, *SD* standard deviation, *XVAS* xerostomic visual analog scale.

*p < 0.05 indicates statistical significance between “Dry mouth” and “Without dry mouth” patients (bold numbers) evaluated using Fisher’s Exact Test or Mann–Whitney U Test.

**Table 2:** Chronic conditions.

	Total no. participants (N = 185)	“Without dry mouth” (n = 66)	“With dry mouth” (n = 119)	*p* value *
**Number of patients, n (%)**				
Cardiovascular disease	71 (44)	25 (38)	43 (48)	**0.025**
Bleeding disorder	6 (4)	1 (2)	4 (5)	**0.030**
Kidney disease	5 (3)	1 (2)	4 (5)	**0.030**
Rheumatoid arthritis	7 (5)	4 (6)	2 (2)	**0.019**
Epilepsy	4 (3)	1 (2)	3 (4)	0.132
Depression	16 (10)	4 (6)	11 (13)	**0.005**
Liver disease	9 (5)	1 (2)	8 (8)	**< 0.001**
Thyroid disease	33 (21)	14 (21)	17 (20)	0.665
Diabetes	16 (10)	3 (5)	12 (14)	**< 0.001**
Dementia	25 (13)	9 (14)	11 (9)	0.070
Lung disease	8 (5)	2 (3)	6 (7)	**0.029**
Stroke	12 (8)	6 (9)	6 (7)	0.343
Gastrointestinal disorder	12 (8)	3 (5)	9 (11)	**0.006**
Tumor disease	22 (13)	5 (8)	16 (16)	**0.001**
Parkinson’s diseases	8 (5)	1 (2)	7 (7)	**0.001**

*p < 0.05 indicates statistical significance between “Dry mouth” and “Without dry mouth” patients (bold numbers) evaluated using Fisher’s Exact Test or Mann–Whitney U Test.

### Impact of dry mouth on smell and taste

There was no difference between “dry mouth” and “without dry mouth” patients regarding identification of odors using the Sniffin’ Sticks or taste using the Taste Strips (*p* > 0.05). The odor that was most often identified correctly was clove (86% of patients), while the odor that was least identified correctly was lemon (49%). The taste that was most often identified correctly was sweet (88%), while the taste that was least identified correctly was sour (75%) (Table 3).

**Table 3:** Identification of smells (Sniffin’ Sticks) and tastes (Taste Strips) according to dry mouth status.

	Study participants (N = 185)	“Without dry mouth” (n = 66)	“With dry mouth” (n = 119)	*p* value*
**Number of patients, n (%)**				
Correctly recognized smell				
Orange	155 (83.8)	56 (84.8)	99 (83.2)	0.837
Leather	137 (74.1)	49 (74.2)	88 (73.9)	1.000
Cinnamon	128 (69.2)	49 (74.2)	79 (66.4)	0.320
Peppermint	154 (83.2)	54 (81.8)	100 (84)	0.687
Banana	135 (73)	47 (71.2)	88 (73.9)	0.731
Lemon	90 (48.6)	35 (53)	55 (46.2)	0.443
Licorice	135 (73)	49 (74.2)	86 (72.3)	0.863
Coffee	148 (80)	55 (83.3)	93 (78.2)	0.448
Clove	159 (85.9)	55 (83.3)	104 (87.4)	0.509
Pineapple	109 (58.9)	41 (62.1)	68 (57.1)	0.536
Rose	146 (78.9)	55 (83.3)	91 (76.5)	0.348
Fish	154 (83.2)	56 (84.8)	98 (82.4)	0.837
Correctly recognized taste				
Salty	149 (80.5)	55 (83.3)	94 (79)	0.563
Sweet	163 (88.1)	57 (86.4)	106 (89.1)	0.638
Bitter	154 (83.2)	52 (78.8)	102 (85.7)	0.304
Sour	139 (75.1)	48 (72.7)	91 (76.5)	0.597
**Mean (SD) or median (IQR)**				
Correctly identified odors, n	9 (3.1)	9.2 (3.1)	8.9 (3.1)	0.310
	10 (8–11)	10 (8–11)	10 (8–11)	
Correctly identified tastes, n	3.3 (1)	3.2 (1.2)	3.3 (1)	0.997
	4 (3–4)	4 (3–4)	4 (3–4)	
Correctly identified tastes plus odors, n	12.2 (3.7)	12.4 (4)	12.1 (3.6)	0.262
	14 (10–15)	14 (11–15)	13 (10–15)	

*IQR* interquartile range, *SD* standard deviation.

*p < 0.05 indicates statistical significance between “Dry mouth” and “Without dry mouth” patients (bold numbers), evaluated using Fisher’s Exact Test or Mann–Whitney U Test.

### Impact of care need on smell and taste

When participants were classified according to “with care need” (n = 41) and “without care need” (n = 149), many significant differences in general and oral health in favor of “without care need” were observed (*p* < 0.001), including younger age, fewer chronic diseases, and more prescribed medications (Table 4). There were no differences regarding whole stimulated (*p* = 0.823) and unstimulated (*p* = 0.668) flow rates, and xerostomic VAS (*p* = 0.490). However, there was a significant difference in the number of correctly identified odors and tastes in favor of the “without care need” patients (Table 4). In the “with care need” group, the odor that was most often identified correctly was fish (46%), while the odor that was least correctly identified was pineapple (24%). Salty, sweet and bitter tastes were identified correctly equally often in this group (59–61%).

**Table 4:** Identification of smells (Sniffin’ Sticks) and tastes (taste strips) according to care need (nursing grade).

	Study participants (N = 190)	Without care need (n = 149)	With care need (n = 41)	*p* value*
**Number of patients, n (%)**				
Gender, female	108 (56.8)	78 (52.3)	30 (73.2)	**0.021**
Periodontitis (CPITN)	46 (59)	17 (42.5)	29 (76.3)	**0.003**
Hyposalivation				
Unstimulated	56 (31.6)	48 (32.4)	8 (27.6)	0.668
Stimulated	63 (36.6)	53 (36.1)	10 (40)	0.823
Correctly recognized smell				
Orange	156 (82.1)	141 (94.6)	15 (36.6)	**< 0.001**
Leather	137 (72.1)	119 (79.9)	18 (43.9)	**< 0.001**
Cinnamon	128 (67.4)	115 (77.2)	13 (31.7)	**< 0.001**
Peppermint	155 (81.6)	140 (94)	15 (36.6)	**< 0.001**
Banana	135 (71.1)	120 (80.5)	15 (36.6)	**< 0.001**
Lemon	91 (47.9)	78 (52.3)	13 (31.7)	**0.022**
Licorice	136 (71.6)	125 (83.9)	11 (26.8)	**< 0.001**
Coffee	149 (78.4)	135 (90.6)	14 (34.1)	**< 0.001**
Clove	159 (83.7)	142 (95.3)	17 (41.5)	**< 0.001**
Pineapple	109 (57.4)	99 (66.4)	10 (24.4)	**< 0.001**
Rose	146 (76.8)	132 (88.6)	14 (34.1)	**< 0.001**
Fish	155 (81.6)	136 (91.3)	19 (46.3)	**< 0.001**
Correctly recognized taste				
Salty	150 (78.9)	126 (84.6)	24 (58.5)	**0.001**
Sweet	164 (86.3)	139 (93.3)	25 (61)	**< 0.001**
Bitter	155 (81.6)	130 (87.2)	25 (61)	**< 0.001**
Sour	139 (73.2)	122 (81.9)	17 (41.5)	**< 0.001**
**Mean (SD) or median (IQR)**				
Age, years	66.7 (15.2)	62.5 (13.8)	81.9 (9.7)	**< 0.001**
	67.5 (57–78)	63 (55–72)	84 (77–87)	
Total API, n	3.4 (3.5)	2.3 (2.5)	7.4 (3.7)	**< 0.001**
	2 (1–5)	2 (0–4)	7 (5–10)	
Chronic diseases, n	2.6 (2.6)	1.7 (1.6)	6 (2.7)	**< 0.001**
	2 (1–4)	1 (1–2)	6 (4–8)	
BMI	25.4 (4.6)	25.3 (4.6)	25.6 (4.7)	0.511
	25 (22–28)	25 (22–28)	25 (23–28)	
DMFT	18.7 (6.4)	17.3 (6.4)	22.2 (4.9)	**< 0.001**
	20 (15–23)	18 (13.5–22)	23 (19–28)	
Root caries index	0.3 (0.7)	0.1 (0.2)	1 (1.2)	**< 0.001**
	0 (0–0.2)	0 (0–0)	0.6 (0.2–1.5)	
XVAS	3.8 (3.3)	3.9 (3.4)	3.5 (3)	0.490
	4 (0–7)	4 (0–7)	3.5 (0–5)	
Salivary flow rate, ml/min				
Unstimulated	0.4 (0.4)	0.4 (0.3)	0.4 (0.5)	0.789
	0.3 (0.2–0.5)	0.3 (0.2–0.6)	0.3 (0.1–0.5)	
Stimulated	1.2 (0.9)	1.2 (0.9)	1 (0.8)	0.516
	1 (0.4–1.6)	1.1 (0.4–1.7)	0.8 (0.5–1.6)	
Correctly identified odors, n	8.8 (3.3)	10 (2.1)	4.4 (3.2)	**< 0.001**
	10 (8–11)	11 (9–11)	4 (2–6)	
Correctly identified tastes, n	3.2 (1.1)	3.5 (0.8)	2.2 (1.5)	**< 0.001**
	4 (3–4)	4 (3–4)	3 (1–3)	
Correctly identified odors plus tastes, n	11.9 (4.1)	13.4 (2.4)	6.5 (4.3)	**< 0.001**
	13 (10–15)	14 (13–15)	7 (3–10)	

*API* active pharmaceutical ingredients, *BMI* body mass index, *CPITN* community periodontal index of treatment needs, *DMFT* decayed missed filled teeth, *IQR* interquartile range, *SD* standard deviation, *XVAS* xerostomic visual analog scale.

*p < 0.05 indicates statistical significance (bold numbers), evaluated using Fisher’s Exact Test or Mann–Whitney U Test.

### Regression analysis

The ability to identify odors and tastes correctly was influenced by age (*p* < 0.001), the number of prescribed medications (*p* < 0.001 and *p* = 0.01, respectively), and the number of chronic diseases (*p* < 0.001 and *p* = 0.01). xerostomic VAS, gender, and stimulated or unstimulated hyposalivation did not have an impact on the recognition of odors or tastes. Results of the univariate binary logistic regression analysis of single odors also revealed the influence of age, number of prescribed medications, and number of chronic diseases for all odors (*p* < 0.05).

There was an impact on taste recognition according to: xerostomic VAS (*p* = 0.032) and age (*p* < 0.001) for the salty taste; age (*p* = 0.002), number of medications (*p* = 0.003), and number of chronic diseases (*p* = 0.006) for the sweet taste; age (*p* = 0.035) and gender (*p* = 0.004) for the bitter taste; age (*p* = 0.001), number of medications (*p* < 0.001), and number of chronic diseases (*p* < 0.001) for the sour taste.

## Discussion

We were unable to determine any differences regarding the ability to correctly identify different odors or tastes between patients “with dry mouth” (hyposalivation and/or xerostomia) or “without dry mouth”. In contrast, the need for care was identified as a risk indicator for impaired ability to recognize smells and tastes, which applied to all tastes and smells examined. Furthermore, the ability to identify smells and tastes was negatively influenced by age, the number of medications taken, and the number of chronic comorbidities.

In interpreting these results regarding identification of different odors and tastes in the “with dry mouth” group, it must be noted although this group reported high xerostomia scores, there were no hyposalivation values in the objective unstimulated or stimulated salivary flow rates. These values should have been below the threshold rates described in the literature (unstimulated whole saliva < 0.1 ml/min; stimulated whole saliva < 0.5–0.7 ml/min) to allow inclusion of people with objective hyposalivation^32–34^. Therefore, using our analysis it was not possible to definitively state that objectively-assessed hyposalivation did not serve as a risk factor for taste or smell disorders. However, we were able to conclude that xerostomia alone (the subjective feeling of a dry mouth) did not result in taste or smell disorders.

As confirmed in our study, aging is a risk factors for impaired smell and taste among people with and without chronic diseases. In addition, chronic illnesses and medication use are both risk factors for smell and taste impairment^35^. Causes include increased threshold values for taste and smell, reduced intensity of suprathreshold stimuli, diminished discrimination ability, diminished identification ability, and distorted taste or smell; however, the exact causes of changes in patients with full health and not taking medications have not yet been fully clarified.

Considering the results of the examined oral health, the results for periodontitis and root caries are significantly worse in the patient group with dry mouth. We do not think there is a plausible causal influence of these reduced oral health parameters on a restricted ability to smell or taste; rather, they are to be assessed as well-known and plausible consequences of reduced saliva flow rates as described in the literature^36,37^. In addition, chronic diseases could be identified in the study participants that individually or in combination are known to increase the risk of dry mouth, either through the disease itself or through the associated medication^1^. These diseases include cardiovascular disease, rheumatoid arthritis, depression, diabetes, and Parkinson's disease, among others. In contrast, such a difference could not be shown in patients with lung diseases, epilepsy, or post-stroke condition, and are not described in the literature. Both polypharmacy and the presence of many chronic underlying diseases are also per se risk factors for dry mouth. Since the need for long-term care as a risk factor is a factor that is composed of a vulnerable state of many chronic diseases, the need for care appears to be a suitable representative factor for characterizing such a patient population.

Our findings identify the need for care as a risk indicator for changes in taste and smell, which should be considered alongside the chronic diseases and polypharmacy that classically characterize this group of patients^38^. Taste and smell changes in this group lead to poor appetite^39^, different food choices^40^, and a decrease in nutritional quality^41^. These factors have a broad impact by negatively contributing to disease status, weight loss, and immune competency^17,42–44^, and play a role in the daily management of people who need care. Studies in cancer patients have demonstrated the vicious circle in which these problems contribute to each other over time. Reductions in taste and smell lead to malnutrition and weight loss^45–47^, which might impair patient response to cancer therapies and potentially lead to higher mortality rates^48,49^. We assume that this train of events would also apply to people in need of care, where a functioning immune system is essential to maintain health and wellbeing. In a nursing home setting, in which a third party is responsible for the choice of food and its preparation, the issues surrounding potential impairments in taste and smell and their impact on nutritional status are rarely considered. While parenteral nutrition is used to overcome these problems in cancer or palliative patients towards the end of life, eating a meal independently appears to be highly relevant for patients in need of care, contributing to an improved nutritional status and representing participation in social life. A few studies have evaluated the extent to which flavor-enhanced foods can help to compensate for the loss of taste and smell in nursing home residents, and found that flavor enhancement improved nutritional intake and led to improved immune competence^50–52^. In these studies, simulated food flavors were added to healthy foods such as vegetables and meat and were associated with increased lymphocyte counts, improved functional status, and improved quality of life. Corresponding improvements in taste led to increased salivation with increased IgA secretion^51^. In dentistry, these considerations should also be applied to the selection of oral hygiene products (toothpaste, mouth rinse solution, mouth gels for symptomatic dry mouth relief), where a positive olfactory and taste sensation that is subjectively perceived by people contributes to frequent use. In the literature, it has been reported that with increasing age, the perception threshold seems to increase for sour and bitter tastes, but also salty, sweet, and umami tastes. Contrary to the logical first assumption, the consequence does not seem to be a preference for stronger flavors but rather leads to a shift towards sweet and salty foods^53^. Therefore, oral care products developed especially for older people might be useful, whose tastes appear to be sweet or salty with neutral pH values. In palliative care, the patient's known taste preferences are often considered when alleviating the symptoms of frequently occurring, very dry mouth; cotton swabs are moistened with the patient's favorite drink and placed on the lips and mucous membranes^54^. Since the preferences of older people often focus on rather sweet tastes, oral care products could also include these flavors—not sweetened with sugar, but instead possibly caries-inhibiting substances. Future studies should examine taste preferences, especially among seniors with dry mouth, so that these preferences could be included in the development of oral care products.

Our study showed that subjective dry mouth is not a clear risk indicator for the development of impaired smell and taste in older age. Additional interventions should be implemented to improve the smell and taste of food and stimulate the remaining saliva production, thus optimizing food intake and the use of oral hygiene products.

### Limitations

The main limitation of our study is the retrospective data analysis. However, patients only were included if they had been involved in any form of dry mouth examination. By carefully discussing and describing the study population, we hope to clarify the scientific value of this study. Another limiting factor is the fact that in a very inhomogeneous study population as in this study, a high percentage of the elders in particular are affected by chronic diseases that are associated with a restricted sense of smell or taste (e.g. Parkinson's disease). However, due to the unambiguity of the data, we have no indications that repeating the study in other subgroups would lead to different results. Also, in this retrospective setting we cannot completely rule out that symptom-relieving agents were taken prior to the examination appointments; however, when examination took place, patients were asked about this and no events were reported. In addition, stratification of the “with dry mouth” group into different subpopulations (i.e., unstimulated and stimulated hyposalivation or xerostomia) might have been useful. Unfortunately, as in clinical practice, the individual symptoms of stimulated and unstimulated hyposalivation could not be assessed as individual factors because patients comprise different and unique combinations. For future prospective studies, however, a precise and differentiated assessment of the subjective and objective salivary parameters prior to inclusion in the study, with clearly defined inclusion and exclusion criteria regarding objective and subjective dry mouth values, should be carried out.

## Conclusion

The ability to identify smells and tastes was negatively influenced by age, the number of medications taken, and the number of chronic comorbidities, but subjective dry mouth problems had no impact. In addition, the need for care was identified as an independent risk indicator for impaired ability to recognize smells and tastes. Since a limited ability to smell and taste has a wide negative influence on nutrition, especially in the group of older people with care needs, future focus should be on the development of interventions that positively influence smell and taste. This may also help to increase the flow of saliva, which can in turn contribute to improved nutritional status and improve general health and wellbeing.
